# Blood biochemical status of deep-sea sharks following longline capture in the Gulf of Mexico

**DOI:** 10.1093/conphys/coaa113

**Published:** 2021-01-15

**Authors:** Bianca K Prohaska, Brendan S Talwar, R Dean Grubbs

**Affiliations:** 1 Coastal and Marine Laboratory, Florida State University, St. Teresa, FL 32358, USA; 2 Resource Assessment and Conservation Engineering Division, NOAA Alaska Fisheries Science Center, Seattle, WA 98115, USA; 3 Exuma Sound Ecosystem Research Project, Cape Eleuthera Institute, Eleuthera, The Bahamas; 4 Institute of Environment, Department of Biological Sciences, Florida International University, North Miami, FL 33181, USA

**Keywords:** Stress, elasmobranch, oil spill

## Abstract

Prior to the Deepwater Horizon (DWH) oil spill, little research effort was focused on studying deep-sea sharks in the Gulf of Mexico (GoM). While the biology of these fishes remains virtually unknown, they are routinely captured in commercial fisheries as bycatch. In the absence of basic biological data, and with the probability of post-release survival unknown for most species, effective management plans cannot be formulated, making populations highly susceptible to overfishing. Any potential detrimental effects of the DWH oil spill, which occurred at 1500 m deep, are also unknown. Following longline capture, we characterized the physiological blood biochemical parameters related to secondary stress and compared them among seven shark species occurring on the continental shelf edge and slope in the GoM at depths ranging from 200 to 2000 m*.* We also investigated the relationship between blood parameters and depth as well as proximity to the oil spill site. The deep-sea sharks examined here exhibited variability in blood chemistry associated with the secondary stress response, with values falling within published records for previously studied elasmobranchs. Results suggested that there is greater relative physiological stress in shallower-dwelling sharks as well as smaller-bodied sharks. Further, the rate of core temperature warming was fastest in smaller bodied sharks, which likely contributes to greater physiological stress. The core temperatures of the larger-bodied, deeper-dwelling species were not altered as drastically as the smaller-bodied sharks after being hauled to the surface. Any chronic physiological effects of the oil spill were not detectable as there were no relevant correlations between blood chemistry metrics and proximity to the DWH oil spill site.

## Teaser

Results of this study suggest that deep-sea sharks exhibit variable levels of physiological stress to longline capture. Shallower-dwelling and smaller-bodied sharks exhibited greater apparent physiological disruption. No increase in physiological disruption was detected with proximity of capture to the Deepwater Horizon oil spill site.

## Introduction

Elasmobranchs, the sharks and rays, are captured worldwide in many commercial and recreational fisheries, and ~18.8% of the group’s ~1250 extant species are designated as ‘threatened’ in the International Union for the Conservation of Nature (IUCN) Red List of Threatened Species (IUCN 2019). While a limited number of species are targeted, many are caught as bycatch and released because of low commercial value or fisheries regulations that limit their retention. Unfortunately, many individuals that are released as bycatch are either already dead, die post-release or suffer detrimental long-term sublethal effects from the capture event (e.g. Cooke *et al.* 2002; Morgan and Burgess 2007; Skomal 2007; Morgan *et al.* 2009; Skomal and Mandelman 2012; Brooks *et al.* 2015; Talwar *et al.* 2017). The life history of this group of fishes is generally characterized by slow growth, late maturity, a long lifespan and low fecundity, all characteristics that are even more extreme in deep-sea elasmobranchs (Simpfendorfer and Kyne 2009). These life history characteristics as well as increasing direct and indirect fishing pressures (Morato *et al.* 2006; Cotton and Grubbs 2015; Oliver *et al.* 2015) make elasmobranchs, particularly deep-sea elasmobranchs, susceptible to overexploitation worldwide (Bonfil 1994; Walker 1998; Dulvy *et al.* 2003).

Deep-sea sharks in the Gulf of Mexico (GoM), particularly *Mustelus* spp. and *Squalus* spp., are subject to bycatch and potential post-release mortality as they are captured and discarded in the deep reef-fish longline fisheries (Hale *et al.* 2011; Scott-Denton *et al.* 2011; Scott-Denton and Williams 2013) and shrimp trawl fisheries (Zhang *et al.* 2015). While *Mustelus* spp. are not currently directly harvested in the GoM, they are in the US Atlantic commercial shark fishery, with greater than 900 000 kg whole weight landed in 2012 (Cortés and Balchowsky 2015) and they make up the overwhelming majority of landed shark fins when examining both the small and large coastal shark complexes from 2016 to 2018 (NOAA Fisheries 2020). As traditional fisheries become overfished, the industry will begin to explore new and different habitats, like those in the deep ocean, which will also be fueled by the development of new technology (Cotton and Grubbs 2015). A better understanding of deep-sea taxa before they become targeted would allow for better management and conservation plans from the outset of these fisheries.

Understanding physiology is essential because physiology dictates the life history, behavior and fitness of an organism (Ricklefs and Wikelski 2002; Wikelski and Cooke 2006), and this information can be used in the development or adjustment of species-specific management and conservation plans (Ferguson and Tufts 1992; Wikelski and Cooke 2006; Young *et al.* 2006). For example, corticosterone levels in marine iguanas *Amblyrhynchus cristatus* in the Galapagos were studied during normal years and during El Niño years to predict overall population health and survival rates (Romero and Wikelski 2001). These data became particularly useful when an oil spill occurred in 2001, as researchers were able to predict oil spill-related mortality based on post-oil spill corticosterone levels (Wikelski *et al.* 2001). Previous work across a wide range of taxa including invertebrates and vertebrates has suggested that, in some cases, even closely related species can differ greatly in aspects of their physiology (e.g. Navas 1996; Tomanek and Somero 1999; Hoffman *et al.* 2000). This may give the species with the more robust physiological response to a stressor an advantage over its congener and may help define a species’ ecological niche and range. Congeneric differences have been observed in physiological markers of the stress response in several species of sharks (Manire *et al.* 2001; Mandelman and Skomal 2009; Gallagher *et al.* 2014; Butcher *et al.* 2015), though no comparable work has been done on deep-sea taxa. However, two studies have investigated mortality from longline capture among multiple deep-sea sharks (Brooks *et al.* 2015; Talwar *et al.* 2017).

Stress has been defined in many contexts, but a current working definition is a disruption of homeostasis of an organism by intrinsic or extrinsic stimuli, which can elicit a behavioral or physiological compensatory response (Wendelaar-Bonga 1997). Generally, there are metabolic costs associated with a stress response, and energy is reallocated from growth and reproduction toward respiration, locomotion and tissue repair (Wendelaar-Bonga 1997). Boonstra (2013) notes that the ecology of stress plays an essential role toward understanding a species’ distribution and abundance. In elasmobranchs, it has been suggested that responses are species specific rather than universal (Beerkircher *et al.* 2002; Morgan and Burgess 2007; Mandelman and Skomal 2009; Morgan and Carlson 2010; Hyatt *et al.* 2012; Marshall *et al.* 2012; Skomal and Mandelman 2012; Gallagher *et al.* 2014; Jerome *et al.* 2018), and they are likely influenced by many factors including capture method, capture duration, respiratory mode and metabolic scope (Skomal and Mandelman 2012; Dapp *et al.* 2016; Guida *et al.* 2016; Mohan *et al.* 2020). This response is often measured through physiological changes in blood chemistry (Skomal and Bernal 2010), such as glucose, lactate and acid–base status, which can determine the relative condition of the fish following a stressor such as capture (Cliff and Thurman 1984; Wells *et al.* 1986; Harrenstien *et al.* 2005; Skomal 2007).

The stress response in fishes can broadly be described by the primary (neuroendocrine), secondary (biochemical changes) and tertiary (fitness-related consequences) responses (Skomal and Mandelman 2012), of which the secondary response is the most widely studied in elasmobranchs. Briefly, this response involves numerous changes in blood chemistry that can be easily measured. The concentration of blood glucose is quantified as a proxy of the glucocorticoid hormone stress response, during which gluconeogenesis occurs and reserves of hepatic glycogen are converted to glucose and released to fuel muscle tissue (Barton and Iwama 1991; Hoffmayer and Parsons 2001). Elevated blood glucose, or hyperglycemia, has been observed in elasmobranchs in response to various stressors (Cliff and Thurman 1984; Hoffmayer and Parsons 2001; Skomal 2006; Frick *et al.* 2010). Generally, the partial pressure of carbon dioxide in the blood will also increase in fishes as a result of stressors that cause restricted ventilation, leading to respiratory acidosis, and can be inferred by a decline in blood pH. This can occur in a ram ventilating shark being captured on a line, for instance, or any elasmobranch being entangled in a gillnet (Manire *et al.* 2001; Mandelman and Farrington 2007). Elevated blood lactate leading to metabolic acidosis can occur when an animal switches from aerobic to anaerobic respiration in white muscle tissue as a result of increased energetic demands, such as when evading a predator. This results in a shift of lactate and H+ ions from the muscle to the blood (Black 1958; Schmidt-Nielsen 1997; Skomal 2007) and has been observed in elasmobranchs that are subjected to stress (Murdaugh Jr *et al.* 1965; Rasmussen and Rasmussen 1967; Piiper and Baumgarten 1969; Piiper *et al.* 1972; Martini 1974; Cliff and Thurman 1984). Hyperkalemia, an accumulation of potassium in the blood, has also been observed in elasmobranchs that have endured stress (Cliff and Thurman 1984; Wells *et al.* 1986; Manire *et al.* 2001) and can ensue from intracellular acidosis and the resulting shift of potassium from the muscle to the blood (Cliff and Thurman 1984; Moyes *et al.* 2006). This shift in potassium can modify muscle cell membrane excitability (Adams and Galvan 1986), which can result in myocardial dysfunction in spiny dogfish *Squalus acanthias* (Martini 1974) and muscle tetany in gummy sharks *Mustelus antarcticus* (Cliff and Thurman 1984; Frick *et al.* 2010). Shifts in serum ion concentrations may result in a compensatory mechanism called hemoconcentration (Piiper *et al.* 1972), where fluid shifts from the blood to the muscle to mitigate elevated lactate. Hemoconcentration can also be attributed to red blood cell swelling that increases blood oxygen carrying capacity (McDonald and Milligan 1992) or from catecholamine activation (Nikinmaa 1992). Hemoconcentration has been observed in spiny dogfish*S. acanthias* after trawl capture (Mandelman and Farrington 2007), in lemon sharks *Negaprion brevirostris* following exercise (Bushnell *et al.* 1982) and in capture-stressed shortfin mako sharks *Isurus oxyrinchus* (Wells and Davie 1985), sandbar sharks *Carcharhinus plumbeus* (Brill *et al.* 2008) and smalltooth sawfish *Pristis pectinata* (Prohaska *et al.* 2018). Some of the parameters described above can be indicators of immediate and post-release mortality or post-release behavior (Hoffmayer and Parsons 2001; Young *et al.* 2006; Arlinghaus *et al.* 2009; Talwar *et al.* 2017).

To accurately interpret the relative levels of physiological stress among individuals or taxa, baseline values of commonly assessed metrics are helpful (Skomal and Mandelman 2012). These reference points are uncommon because sharks are difficult to maintain and sample in captivity without causing some degree of stress through the capture and handling process. In the wild, sharks have been sampled after being placed into tonic immobility (Brooks *et al.* 2012) or sampled after limited capture durations in an attempt to provide ‘minimally stressed’ levels (Skomal and Mandelman 2012) as an alternative to absolute baselines. These ‘minimally stressed’ values have not been quantified in any deep-sea sharks, and measuring capture duration at depth is logistically difficult, making it difficult to characterize their physiological stress response to capture along that gradient. Conversely, blood chemistry parameters measured when animals become moribund are often reported in studies focused on stress and mortality in sharks (Moyes *et al.* 2006; Hight *et al.* 2007; Hutchinson *et al.* 2015; Wosnick *et al.* 2017). These values can provide useful end points to help researchers gauge how close an animal is to death as well as put ‘stressed’ values into context by providing an upper bound (Wosnick *et al.* 2017).

**Figure 1:**
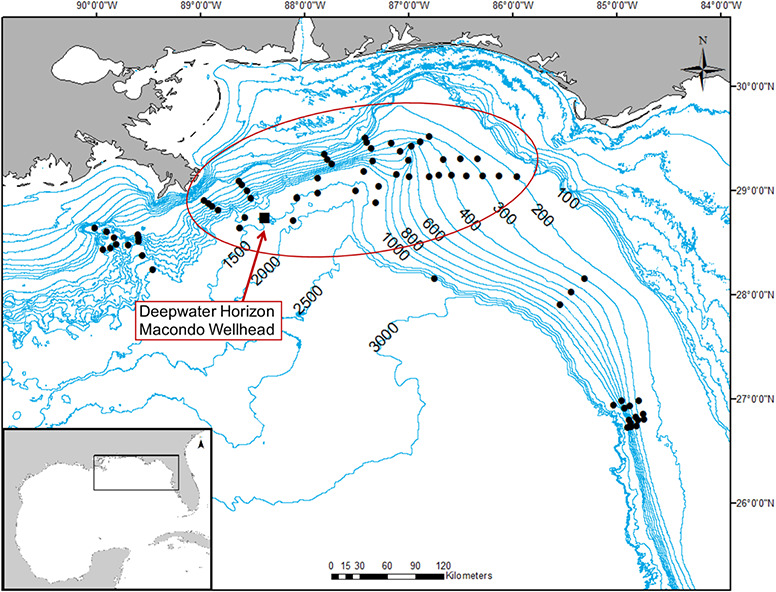
Map highlighting the locations of the fixed survey stations that were sampled during each research cruise, each black dot represents a station that was sampled during the larger survey, while the stations circled in red were locations where samples were collected for this study. The location of the DWH Macondo Wellhead is indicated by a black square with a red arrow.

Given this inherent vulnerability of deep-sea sharks to fisheries (Dulvy *et al.* 2003; Simpfendorfer and Kyne 2009), as well as our limited knowledge of their biology, understanding the effects of extrinsic stressors on these animals is critical, particularly for those that can lead to animal or population-level consequences like major oil spills (Pulster *et al.* 2020). The Deepwater Horizon (DWH) oil spill of 2010 represents one such event that could have unknown effects on deep-sea elasmobranchs. Metabolites of polycyclic aromatic hydrocarbons (PAHs) are commonly studied to identify oil exposure in vertebrates (Gorbi and Regoli 2004; Ferreira *et al.* 2006), and, after the DWH oil spill, Leary (2015) found that exposure to PAHs in deep-sea sharks was highest close to the site of the spill and largely limited to within 100 km of the blowout (Fig. 1). Otherwise, the effects of the DWH oil spill on deep-sea sharks remain to be studied.

The overarching objective of this study was to elucidate the species-specific capture-induced stress physiology of the seven most commonly captured sharks on a deep sea, fishery-independent survey in the GoM, which include the Gulf smooth-hound *Mustelus sinusmexicanus*, the dusky smooth-hound *Mustelus canis*, the Cuban dogfish *Squalus cubensis*, Genie’s dogfish *Squalus clarkae*, the little gulper shark *Centrophorus uyato*, the gulper shark *Centrophorus granulosus* and the bluntnose sixgill shark *Hexanchus griseus.* While we were not able to measure the specific capture duration of each individual sampled and baseline values for blood chemistry parameters are unavailable for these species, all animals were captured in the same manner, allowing us to investigate if observed differences between these species were mediated by habitat, body size, taxonomy (e.g. differences between species, genera or orders) or capture method, to inform fisheries conservation and management plans. Further, this study investigated differences in blood chemistry parameters relative to distance from the DWH oil spill to provide insights into the potential effects of oil spills on elasmobranch physiology.

## Materials and methods

Deep-sea sharks were captured in the GoM and sampled during research cruises conducted by the Florida State University (FSU) aboard the R/V Apalachee. Each cruise consisted of 10–16 days as sea, and blood samples were collected during cruises in June and July 2014, October 2014, March and April 2015, July 2015, September 2015, April and May 2016 and April and May 2017.

### Survey

Modified demersal longlines were used to capture fishes, and two or three longlines were deployed at a time in succession with targeted bottom soak times of 3–4 h. Each longline was ~550 m of 6.4 mm tarred, twisted, hard-laid nylon mainline set on the bottom and consisted of an anchor, followed by a baited chevron trap (60 cm × 60 cm × 38 cm with 2.5 cm mesh), then 50 circle hooks of five different sizes, ranging from 10/0 to 18/0 and spaced 10 m apart, followed by a baited hagfish trap (60 cm × 35 cm with 1.0 cm mesh), a 2-kg sash weight and a Lotek LAT2000 temperature-depth recorder (TDR), followed by scope line to a surface buoy and a strobed highflier. Bottom soak time was calculated as the time between the TDR reaching the bottom during a set and leaving the bottom upon retrieval. Based on average sinking rates of the gear, as measured by the TDR, the time between setting and retrieving the gear at each station was adjusted so that the time the gear soaked on the bottom was approximately the same across all depths and stations. Gear retrieval took ~15 to 45 min depending on the depth of the station. During each cruise, 30–56 demersal longlines were set and hauled at depths ranging from 200 to 2000 m. Sets were made at standard fixed stations located on the continental slope of the eastern GoM from the North Florida Slope across DeSoto Canyon to the Louisiana Slope, with varying distance (18–419 km) from the site of the DWH oil spill (Fig. 1).

### Sampling

As soon as a live shark was landed, a core temperature was immediately assessed by inserting a 20 cm piercing thermometer (VWR, Radnor, PA) into the thickest portion of the dorsal musculature, which was typically 1 cm to either side of the first dorsal fin. The piercing thermometer was inserted 1.5 to 7 cm into the muscle, depending on the size of the shark, and the thermometer was allowed to stabilize (~15 s) before piercing slightly deeper into the muscle. If the temperature decreased, we let the thermometer stabilize again and repeated a deeper probe; however, if the temperature increased, we would pull back and read the coolest temperature of the muscle. Following a temperature reading, and typically 1 min or less after landing, a 5 ml blood sample was collected by caudal venipuncture using a 16–22 gauge needle attached to a heparinized syringe. Following blood collection, animals were euthanized and comprehensively sampled for ongoing and future studies. To assess lactate and pH, a small aliquot of blood was immediately loaded into a CG4+ cartridge and then inserted into a VetScan i-STAT 1 point of care device (Abaxis Inc., Union City, CA), which has been validated for use in elasmobranchs (Gallagher *et al.* 2010; Harter *et al.* 2015). The i-STAT is commonly used in elasmobranch stress studies to provide relative secondary stress data (e.g. Mandelman and Farrington 2007; Mandelman and Skomal 2009; Marshall *et al.* 2012; Guida *et al.* 2016; Talwar *et al.* 2017; Prohaska *et al.* 2018) and has been used on some of the same deep-sea sharks examined herein (Talwar *et al.* 2017). Glucose was measured using an Accu-Chek glucose meter (Roche Diagnostics, Basel, Switzerland), which has been validated for use on fishes (Cooke *et al.* 2008). Hematocrit was measured in duplicate by filling a capillary tube with the homogenized blood sample, capping one end with clay, and spinning the tube in a hematocrit centrifuge at 15 000 g for 5 min. Hematocrit was determined by calculating the red blood cell percentage of the whole blood volume. The remaining whole blood was then centrifuged at 1800 g for 5 min (Unico, Dayton, NJ). The separated plasma was stored at −20°C.

To quantify associations between morbidity and blood chemistry values in *C. uyato*, one of the most commonly captured species, we sampled blood from six individuals captured on the same longline immediately after they reached the deck. We then placed these individuals in a semi-circular tank with flowing, aerated sea surface water. Animals were monitored visually until they were moribund (determined when spiracle movement was negligible, animals were no longer responsive to touch, and there was no buccal reflex when removed from the water). They were then immediately sampled for blood as described previously. Temperature, dissolved oxygen, oxygen saturation and salinity were measured in the tank every 5–14 min using a handheld water quality meter (YSI Pro2030; Yellow Springs, OH, USA). Water conditions were stable during the trial.

In the laboratory, plasma potassium concentrations were measured using a Single-Channel Digital Flame Photometer (Model 02655-00, Cole-Parmer, Vernon Hills, IL, USA). Each sample was prepared using a 1:100 dilution of plasma to Cole-Parmer diluent. Potassium standards (K^+^: 0.5, 1, 2 and 5 ppm) were prepared with a 1000 ppm stock solution. Potassium ions were measured by running a standard curve (in triplicates) before the samples, which were then measured in triplicates and in groups of five. This process was repeated to ensure proper calibration. Measurement of each standard and sample dilution followed protocol developed by the manufacturer (Cole-Parmer), wherein the standard or sample was aspirated for 20 s prior to recording the concentration. Between each measurement, air was aspirated for 10 s, followed by Cole-Palmer diluent for 20 s and air again for 10 s.

### Statistical analyses

Data for blood pH were temperature corrected to water temperature measurements at the depth of capture (Mandelman and Skomal 2009; Gallagher *et al.* 2010; Harter *et al.* 2015). Because hematocrit is a percentage, those data were arcsine transformed before analysis.

Prior to analyses, a non-metric multidimensional scaling (NMDS) was conducted of all blood chemistry metrics from all sharks sampled. The NMDS was followed by a series of permutational multivariate analyses of variance (PERMANOVA) of the NMDS scores as a function of the following independent variables: depth, species, core temperature change (ΔCT—the difference between capture temperature at depth and muscle temperature at vessel) and distance between the capture site and the DWH oil spill. These preliminary analyses were conducted to identify if one or more dependent variables were correlated. The NMDS plot and stress plot can be found in appendices 1 and 2. The results of the PERMANOVAs indicated that the dependent variables were not correlated and that independent statistical analyses were appropriate (Table 1).

**Table 1:** Results of PERMANOVA on NMDS scores as a function of the species, the depth at which the shark was caught, the change in core temperature (core) and the distance from the DWH oil spill at which the animal was caught (distance)

Parameter	F	DF	*P*-value
Species	26.41	6192	0.001
Depth	93.57	1197	0.001
Core temp	4.67	1161	0.04
Distance	11.26	1197	0.001

One-way analyses of variance (ANOVA) were conducted between each species and their total lengths, capture temperature and capture depth to identify significant inter-specific differences. To investigate inter-specific differences in the physiological parameters glucose, lactate, pH, potassium and hematocrit, an ANOVA, a Welch’s ANOVA or a Kruskal–Wallis test was conducted depending on whether data were normally distributed and homoscedastic. When ANOVAs or their analogs were significant, a Tukey honestly significant difference or Dunn’s test was conducted to identify significant pairwise differences. Because these species inhabit discrete depth ranges, a one-way analysis of co-variance (ANCOVA) was conducted prior to the analyses to investigate inter-specific differences in the physiological parameters while controlling for the covariate ‘depth’; however, on further investigation across species, there were non-overlapping covariate ranges for these parameters and depth, making an ANCOVA an inappropriate analysis for these data.

To investigate how core temperature warming may affect physiological parameters in each species, linear regressions were conducted between each parameter and ΔCT. Linear regressions were also conducted for each species, investigating the relationship between each individual physiological parameter and the distance from the DWH oil spill at which they were captured. When data were not normally distributed, a Kendall’s rank correlation was conducted. A small number of *S. cubensis* (*n* = 9) and *C. uyato* (*n* = 8) were captured in chevron traps, and, to identify significant differences in the physiological disturbance induced by longline versus trap capture methods, *t*-tests or Wilcoxon rank sum tests were conducted depending on data normality and homoscedasticity. To account for multiple comparisons of physiological parameters in our analyses, we calculated a Bonferroni-corrected $\alpha$ of 0.007 for lactate, pH and hematocrit and 0.008 for potassium and glucose when conducting linear regressions, *t*-tests and their non-parametric analogs for consideration when interpreting our results.

## Results

**Figure 2:**
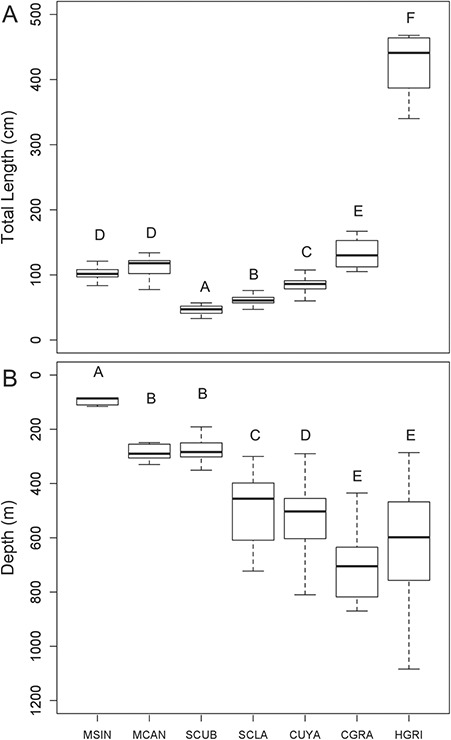
Box plots of (A) total length (cm) and (B) depth (m) of capture of *M. sinusmexicanus* (MSIN), *M. canis* (MCAN), *S. cubensis* (SCUB), *S. clarkae* (SCLA), *C. uyato* (CUYA), *C. granulosus* (CGRA) and *H. griseus* (HGRI), letters above bars indicate significant pairwise differences within each plot.

During the seven research cruises, 265 longline sets were made. The mean soak time was 3.93 h (SD 0.66 h), and soak time did not differ as a function of depth (adjusted *r*^2^ = 0.003, *P* = 0.703). Blood samples were collected for blood chemistry analyses from a total of 450 sharks representing seven species. The catch consisted of 19 *M. sinusmexicanus* (102 ± 2.95 cm; ‘average total length (TL) ± SE’), 17 *M. canis* (111 ± 3.79 cm), 94 *S. cubensis* (47 ± 0.65 cm), 79 *S. clarkae* (61 ± 0.77 cm), 209 *C. uyato* (83 ± 0.94 cm), 21 *C. granulosus* (132 ± 5.09 cm) and 11 *H. griseus* (426 ± 27.09 cm) (Fig. 2A).

These seven shark species varied significantly in both length and capture depth (length: Welch’s ANOVA: *F*_6,56.2_ = 280.72, *P* < 2e-16; depth: Welch’s ANOVA: *F*_6,59.4_ = 327.21,*P* < 2e-16). *M. sinusmexicanus* were caught at the shallowest depths of all species (100 ± 6.98 m; ‘average depth ± SE’). *M. canis* and *S. cubensis* were found at significantly deeper depths than *M. sinusmesicanus*; however, not significantly different from one another at 279 ± 17.95 m and 270 ± 4.79 m, respectively. *S. clarkae* were captured significantly deeper at an average depth of 472 ± 12.77 m. *C. uyato* were found significantly deeper at an average of 535 ± 7.95 m. *C. granulosus* and *H. griseus* were captured significantly deeper yet at average depths of 711 ± 39.45 m and 679 ± 92.73 m, respectively (Fig. 2B). Temperature was negatively correlated with depth; therefore, capture temperature was also significantly different between species (Kruskal–Wallis: *Χ*^2^ = 275.53, df = 6, *P* = 2.2e-16). The warmest average capture temperatures were found where the shallowest species, *M. sinusmexicanus*, was captured (18.24 ± 0.30°C; ‘average temperature ± SE’). Mean capture temperature was slightly cooler at the depth that *M. canis* and *S. cubensis* were caught, with temperatures averaging 13.97 ± 0.49°C and 13.56 ± 0.17°C, respectively. The mean capture temperature for the next deepest dwelling species, *S. clarkae*, was significantly cooler at 9.39 ± 0.28°C. There were no significant differences in mean capture temperatures for *C. uyato* (8.08 ± 0.11°C), *C. granulosus* (7.20 ± 0.50°C), and *H. griseus* (7.72 ± 0.78°C).

### Inter-specific differences in blood chemistry metrics

**Figure 3:**
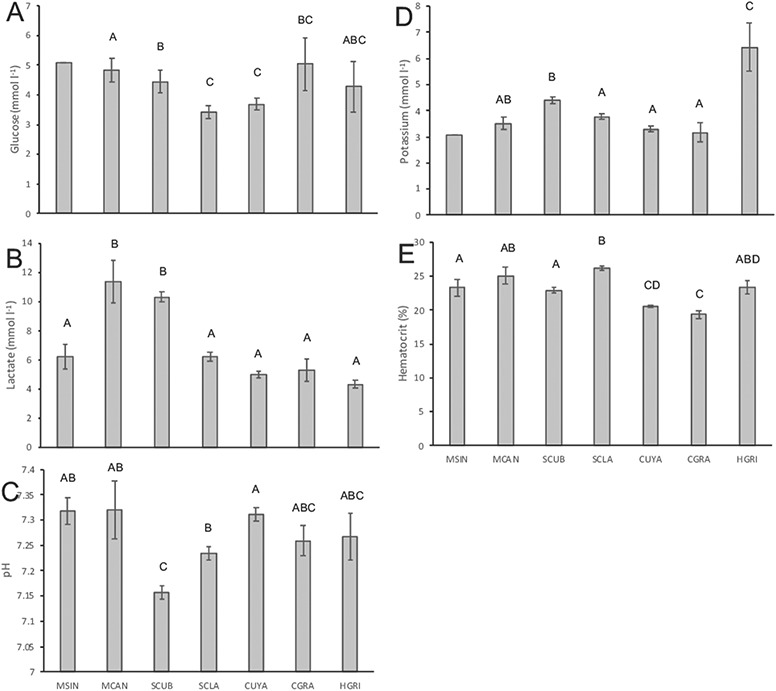
Mean (± SE) of the at-vessel blood chemistry parameters (A) glucose (mmol l^−1^), (B) lactate (mmol l^−1^), (C) pH, (D) potassium (mmol l^−1^) and (E) hematocrit (%) of longline-captured *M. sinusmexicanus* (MSIN), *M. canis* (MCAN), *S. cubensis* (SCUB), *S. clarkae* (SCLA), *C. uyato* (CUYA), *C. granulosus* (CGRA) and *H. griseus* (HGRI), letters indicate significant pairwise differences within each plot.

There were significant differences in median blood glucose concentrations between the six species of sharks for which glucose was analyzed (Kruskal–Wallis: *Χ*^2^ = 40.56, df = 5, *P* = 1.1e-7). *M. canis* had a significantly higher median blood glucose concentration than *S. cubensis*, *S. clarkae*, *C. uyato* and *C. granulosus*, but not *H. griseus*. *S. cubensis* had the next highest median blood glucose concentration, higher than that of *S. clarkae* and *C. uyato*; however, it was not higher than that of *C. granulosus* or *H. griseus*. Lastly, *S. clarkae* and *C. uyato* displayed the lowest median blood glucose concentrations; however, they were not significantly different from that of *C. granulosus* or *H. griseus* (Table 2; Fig. 3A). While *M. sinusmexicanus* was not included in the statistical comparison because of low sample size, this species’ mean blood glucose concentration was high compared to that of other species, and similar to that of *M. canis* (Fig. 3A). These results should be interpreted with caution, as the intraspecific variability in at-vessel blood glucose concentrations was high (Fig. 3A).

**Table 2:** Mean (± SE) at-vessel blood chemistry parameters of longline captured *M. sinusmexicanus* (MSIN), *M. canis* (MCAN), *S. cubensis* (SCUB),*S. clarkae* (SCLA), *C. uyato* (CUYA), *C. granulosus* (CGRA) and *H. griseus* (HGRI)

	Glucose (mmol l^−1^)	Lactate (mmol l^−1^)	pH	Potassium (mmol l^−1^)	Hematocrit (%)
MSIN	5.11 ± 0	6.25 ± 0.86 (11)	7.31 ± 0.03 (11)	3.06 ± 0 (1)	23.32 ± 1.22 (19)
MCAN	4.83 ± 0.41	11.40 ± 1.47 (14)	7.32 ± 0.06 (16)	3.51 ± 0.24 (15)	25.07 ± 1.18 (17)
SCUB	4.45 ± 0.38	10.35 ± 0.38 (62)	7.16 ± 0.01 (63)	4.41 ± 0.13 (81)	22.88 ± 0.40 (92)
SCLA	3.43 ± 0.22	6.22 ± 0.31 (62)	7.23 ± 0.01 (63)	3.77 ± 0.13 (73)	26.15 ± 0.38 (79)
CUYA	3.69 ± 0.21	4.97 ± 0.23 (85)	7.31 ± 0.01 (85)	3.29 ± 0.10 (98)	20.52 ± 0.20 (209)
CUYA^*^	2.90 ± 0.24	11.16 ± 0.35 (6)	7.17 ± 0.04 (6)		23.17 ± 1.19 (6)
CGRA	5.04 ± 0.88	5.30 ± 0.76 (15)	7.26 ± 0.03 (14)	3.16 ± 0.36 (14)	19.35 ± 0.57 (21)
HGRI	4.28 ± 0.85	4.35 ± 0.29 (8)	7.27 ± 0.05 (9)	6.44 ± 0.93 (10)	23.33 ± 0.95 (10)

CUYA^*^ are parameters for at-moribund individuals that were kept in an on-board observation tank. Sample sizes are in parentheses.

There were significant inter-specific differences in mean blood lactate concentrations (Welch’s ANOVA: *F*_6,45.8_ = 31.35, *P* = 1.2e-14). Mean blood lactate was significantly higher in *M. canis* and *S. cubensis* than in the other five sharks analyzed, including *M. sinusmexicanus*, *S. clarkae*, *C. uyato*, *C. granulosus* and *H. griseus* (Table 2; Fig. 3B).

There were significant inter-specific differences in mean blood pH (ANOVA: *F*_6,254_ = 11.53, *P* = 2.08e-11). *S. cubensis* had significantly lower mean blood pH than *M. sinusmexicanus*, *M. canis*, *S. clarkae* and *C. uyato*. *S. clarkae* had the next lowest blood pH, but it was only significantly lower than that of *C. uyato*. Mean blood pH in *C. granulosus* and *H. griseus* was not significantly different than that of any other species (Table 2; Fig. 3C).

There were significant inter-specific differences in mean blood potassium concentrations (ANOVA: *F*_5,285_ = 18.78, *P* = 3.93e-16). *H. griseus* had the highest mean concentration of the six species examined. *S. cubensis* had the next highest potassium concentration; however, it was not significantly higher than that of *M. canis*. The species with the lowest mean blood potassium concentration were *S. clarkae*, *C. uyato* and *C. granulosus*; however, those values were not significantly lower than that of *M. canis*. While the mean blood potassium concentration of *M. sinusmexicanus* was not included in this analysis because of low sample size, it was similar to that of the four shark species with low blood potassium—*M. canis*, *S. clarkae*, *C. uyato* and *C. granulosus* (Table 2; Fig. 3D).

There were significant inter-specific differences in mean hematocrit (ANOVA: *F*_6,440_ = 31.7, *P* < 2e-16). *S. clarkae* had the highest mean hematocrit of any species examined, which was significantly higher than that of *M. sinusmexicanus*, *S. cubensis*, *C. uyato* and *C. granulosus*, but not significantly different than that of *M. canis* or *H. griseus*. *M. sinusmexicanus* and *S. cubensis* had the next highest hematocrit, although it was not significantly different than that of *M. canis* or *H. griseus*, while *C. uyato* and *C. granulosus* had significantly lower hematocrit levels relative to other species (Table 2; Fig. 3E).

### Change in core temperature

Average (± SE) changes between at-vessel core temperature and bottom temperature (ΔCT) were 2.05 ± 0.71°C for *M. sinusmexicanus*, 3.49 ± 0.39°C for *M. canis*, 6.70 ± 0.29°C for *S. cubensis*, 10.30 ± 0.47°C for *S. clarkae*, 6.29 ± 0.19°C for *C. uyato*, 2.31 ± 0.21°C for *C. granulosus* and 3.75 ± 0.92°C for *H. griseus*.

No significant relationships were found between the seven blood chemistry parameters and ΔCT for *M. sinusmexicanus*, *M. canis* or *C. granulosus* (Table 3). In *S. cubensis*, pH significantly decreased (*R*^2^ = 0.14, *P* = 0.01) and hematocrit significantly increased (*R*^2^ = 0.09, *P* = 0.01) with increasing ΔCT. When considering the Bonferroni correction, neither aforementioned parameter significantly changed with ΔCT. Glucose, lactate and potassium did not change with ΔCT in *S. cubensis* (Table 3).

**Table 3:** Results of linear regressions investigating how at-vessel blood chemistry parameters of longline captured *M. sinusmexicanus* (MSIN), *M. canis* (MCAN), *S. cubensis* (SCUB), *S. clarkae* (SCLA), *C. uyato* (CUYA), *C. granulosus* (CGRA) and *H. griseus* (HGRI) change with core temperature

Species	Parameter	P or NP	Test statistics (*F* or *Z*)	DF	*R* ^2^ or tau	*P*-value
MSIN						
	Lactate	P	0.46	1, 1	0.31	0.62
	pH	P	0.91	1, 1	0.48	0.51
	Hct	P	24.06	1, 1	0.96	0.13
MCAN						
	Glucose	P	1.21	1, 8	0.13	0.30
	Lactate	P	1.40	1, 7	0.17	0.28
	pH	P	0.32	1, 7	0.04	0.59
	K	P	0.01	1, 8	1.9e-3	0.91
	Hct	P	0.001	1, 8	8.4e-5	0.98
SCUB						
	Glucose	NP	0.48	1, 67	0.04	0.63
	Lactate	P	0.01	1, 43	1.2e-4	0.94
	pH	P	6.94	1, 43	0.14	0.01 ^*^
	K	P	0.24	1, 68	3.5e-3	0.63
	Hct	P	6.96	1, 72	0.09	0.01 ^*^
SCLA						
	Glucose	NP	2.96	1, 53	0.28	3.1e-3 ^**^
	Lactate	P	2.92	1, 38	0.07	0.10
	pH	P	5.75	1, 39	0.13	0.02 ^*^
	K	P	1.42	1, 54	0.03	0.24
	Hct	P	3.35	1, 54	0.09	0.07
CUYA						
	Glucose	NP	6.57	1, 159	0.35	5.1e-11 ^**^
	Lactate	P	0.003	1, 69	5.0e-5	0.95
	pH	P	1.08	1, 69	0.02	0.30
	K	P	0.28	1, 92	3.0e-3	0.60
	Hct	P	0.44	1, 179	2.5e-3	0.51
CGRA						
	Glucose	P	3.26	1, 10	0.25	0.10
	Lactate	P	0.002	1, 7	3.2e-4	0.96
	pH	P	0.67	1, 8	0.08	0.44
	K	P	0.51	1, 10	0.05	0.49
	HCT	P	0.78	1, 10	0.07	0.40
HGRI						
	Glucose	P	5.45	1, 4	0.58	0.08
	Lactate	P	0.27	1, 4	0.06	0.63
	pH	P	1.38	1, 4	0.26	0.31
	K	P	13.99	1, 3	0.82	0.03 ^*^
	Hct	P	13.05	1, 3	0.81	0.04 ^*^

P denotes a parametric test was conducted, while NP denotes a non-parametric test was conducted. A ^*^ following a *P*-value denotes significance at an $\alpha$ level of 0.05, while ^**^ denotes significance at the Bonferroni-corrected $\alpha$ level.

In *S. clarkae*, there was a significant increase in glucose (tau = 0.28, *P* = 3.1e-3) and a significant decrease in pH (*R*^2^ = 0.13, *P* = 0.02) with increasing ΔCT; however, the change in pH was not significant with the Bonferroni correction. No significant relationships were found between lactate, potassium or hematocrit and ΔCT in *S. clarkae* (Table 3).

In *C. uyato*, a significant increase in glucose (tau = 0.35, *P* = 5.1e-11) was found with increasing ΔCT. No significant changes were observed in the six other physiological parameters relative to ΔCT (Table 3).

In *H. griseus*, hematocrit increased significantly (*R*^2^ = 0.81, *P* = 0.04) while potassium decreased significantly (*R*^2^ = 0.82, *P* = 0.03) with increasing ΔCT. The remaining parameters (glucose, lactate and pH) had no relationship with ΔCT (Table 3). It is important to note that none of these parameters significantly changed with ΔCT when considering the Bonferroni correction.

### Moribund blood chemistry metrics

Six mature gulper sharks *C. uyato* were captured on one longline set for 371 minutes at a maximum depth of 711 m and a minimum temperature of 6.41°C. Because of sea conditions, this set had among the longest soak times of any in the survey. All individuals were hooked in the jaw on 10/0, 12/0 or 14/0 hooks. Once placed in an on-deck tank, each shark floated upside down and buccal pumped and/or respired through its spiracles for 14 to 47 min before becoming moribund. No sharks showed signs of recovery. Blood chemistry metrics for at-moribund sharks appear in Table 2.

### Capture method

Two species, *S. cubensis* and *C. uyato*, were also captured in chevron traps in large enough numbers (albeit fewer than on longlines) to allow for a comparison of physiological parameters between longline- and trap-captured sharks (Table 4). *S. cubensis* had significantly higher lactate after longline capture than after trap capture (Table 5). *C. uyato* had significantly higher blood potassium after trap capture than after longline capture (Table 6). No other blood chemistry parameters were significantly different between capture methods in either species.

**Table 4:** Mean (± SE) at-vessel blood chemistry parameters of trap and longline (LL) captured *S. cubensis* (SCUB) and *C. uyato* (CUYA)

		Glucose (mmol l^−1^)	Lactate (mmol l^−1^)	pH	Potassium (mmol l^−1^)	Hematocrit (%)
SCUB	Trap	4.70 ± 1.72 (9)	5.83 ± 0.39 (9)	6.48 ± 0.03 (9)	4.43 ± 0.55 (4)	25.06 ± 1.44 (8)
	LL	4.79 ± 0.40 (89)	10.42 ± 0.39 (63)	7.16 ± 0.01 (63)	4.41 ± 0.14 (77)	22.38 ± 0.41 (100)
CUYA	Trap	2.78 ± 0.37 (8)	3.60 ± 0.45 (8)	6.48 ± 0.03 (8)	4.60 ± 0.41 (5)	22.13 ± 1.19 (8)
	LL	3.83 ± 0.23 (175)	5.03 ± 0.24 (78)	7.31 ± 0.01 (78)	3.20 ± 0.10 (94)	20.47 ± 0.19 (219)

Sample sizes in parentheses.

**Table 5:** Statistical results of *t*-tests or Wilcoxon rank sum tests comparing at-vessel blood chemistry parameters of *S. cubensis* caught by trap or longline (LL)

	Sample size			Test statistics		
	Trap	LL	P or NP	(T or W)	DF	*P*-value
Glucose	9	89	NP	509	96	0.19
Lactate	9	63	P	3.78	70	3.2e-3
pH	9	63	P	−1.12	70	0.27
K	4	77	P	0.03	79	0.98
Hct	8	100	P	−1.72	106	0.09

P denotes a parametric test was conducted, while NP denotes a non-parametric test was conducted.

**Table 6:** Statistical results of *t*-tests or Wilcoxon rank sum tests comparing at-vessel blood chemistry parameters of *C. uyato* caught by trap or longline (LL)

	Sample size			Test statistics		
	Trap	LL	P or NP	(T or W)	DF	*P*-value
Glucose	8	178	NP	805	181	0.48
Lactate	8	78	NP	391	84	0.25
pH	8	78	P	0.44	84	0.6
K	5	94	P	−3.24	97	0.0016
Hct	8	219	P	−1.59	225	0.11

P denotes a parametric test was conducted, while NP denotes a non-parametric test was conducted.

### Distance from DWH oil spill

No significant relationships were found between the seven blood chemistry parameters and the distance between the DWH oil spill site and the capture site for *M. sinusmexicanus*, *M. canis*, *S. clarkae*, *C. granulosus* or *H. grisius* (Table 7). A significant decrease in hematocrit was found in *S. cubensis* with increasing distance from the DWH oil spill site (Table 7). In *C. uyato*, glucose and potassium significantly increased, while lactate significantly decreased with increasing distance from the DWH oil spill site (Table 7). When considering the Bonferroni correction, the only significant change was the increase in glucose in *C. uyato*.

**Table 7:** Results of linear regressions investigating how at-vessel blood chemistry parameters of longline captured *M. sinusmexicanus* (MSIN), *M. canis* (MCAN), *S. cubensis* (SCUB), *S. clarkae* (SCLA), *C. uyato* (CUYA), *C. granulosus* (CGRA) and *H. griseus* (HGRI) change with distance between the site of the DWH oil spill and the animal capture site

Species	Parameter	P or NP	Test statistics (F or Z)	DF	*R* ^2^ or tau	*P*-value
MSIN						
	Lactate	P	2.77	1, 9	0.24	0.13
	pH	P	5.1e-4	1, 9	5.7e-5	0.98
	Hct	P	1.16	1, 17	0.06	0.30
MCAN						
	Glucose	P	0.85	1, 13	0.06	0.37
	Lactate	P	1.91	1, 12	0.14	0.19
	pH	P	1.41	1, 14	0.09	0.26
	K	P	0.15	1, 13	0.01	0.70
	Hct	P	0.35	1, 15	0.02	0.56
SCUB						
	Glucose	NP	0.81	1, 83	0.07	0.42
	Lactate	P	0.52	1, 60	8.6e-3	0.47
	pH	P	2.11	1, 61	0.03	0.15
	K	P	0.52	1, 79	6.6e-3	0.47
	Hct	P	6.72	1, 90	0.07	0.01 ^*^
SCLA						
	Glucose	NP	0.74	1, 69	0.07	0.46
	Lactate	P	0.02	1, 60	2.7e-4	0.90
	pH	P	2.26	1, 60	0.04	0.14
	K	P	0.09	1, 71	13.e-3	0.76
	Hct	P	3.37	1, 77	0.04	0.07
CUYA						
	Glucose	NP	4.59	1, 179	0.25	4.3e-6 ^**^
	Lactate	P	6.77	1, 83	0.07	0.01 ^*^
	pH	P	3.9e-3	1, 83	4.7e-5	0.95
	K	P	5.87	1, 96	0.06	0.02 ^*^
	Hct	P	3.77	1, 207	0.02	0.05
CGRA						
	Glucose	P	3.49	1, 12	0.23	0.09
	Lactate	P	3.07	1, 13	0.19	0.10
	pH	P	0.19	1, 12	0.02	0.67
	K	P	0.68	1, 12	0.05	0.43
	HCT	P	1.06	1, 19	0.05	0.32
HGRI						
	Glucose	P	1.05	1, 8	0.12	0.34
	Lactate	P	1.13	1, 6	0.16	0.33
	pH	P	4.56	1, 7	0.39	0.07
	K	P	0.02	1, 8	2.8e-3	0.88
	Hct	P	4.12	1, 8	0.34	0.08

P denotes a parametric test was conducted, while NP denotes a non-parametric test was conducted. A ^*^ following a *P*-value denotes significance at an $\alpha$ level of 0.05, while ^**^ denotes significance at the Bonferroni-corrected $\alpha$ level.

## Discussion

This is the third study following Barkley *et al.* (2017) and Talwar *et al.* (2017) to examine blood chemistry after capture in deep-sea sharks. The seven species of sharks examined herein vary in size from the small *S. cubensis* to the large *H. griseus* and also vary in the depths at which they reside, with *M. sinusmexicanus*, *M. canis* and *S. cubensis* inhabiting depths shallower than 300 m and *S. clarkae*, *C. uyato*, *C. granulosus* and *H. griseus* inhabiting depths of about 500 m and greater (Fig. 2B).

We documented variability in blood chemistry values after capture for these seven species of deep-sea shark. In general, values suggested more disturbed blood chemistry than reported for shallower-dwelling demersal elasmobranch species such as the southern stingray *Hypanus americana* (Cain *et al.* 2004), Port Jackson shark *Heterodontus portusjacksoni* (Frick *et al.* 2010) and smalltooth sawfish *P. pectinata* (Prohaska *et al.* 2018). Values were similar to those of gillnet- and longline-captured gummy sharks*M. antarcticus* (Frick *et al.* 2010) and trawl-captured spiny dogfish *S. acanthias* (Mandelman and Farrington 2007). While multiple blood chemistry parameters were similar between the species examined here and some coastal and pelagic sharks studied to date (Marshall *et al.* 2012; Gallagher *et al.* 2014), they generally suggested less physiological disturbance for those individuals examined here than reported for species such as the great hammerhead *Sphyrna mokarran* (Gallagher *et al.* 2014), blacktip *Carcharhinus limbatus*, porbeagle *Lamna nasus* and pelagic thresher *Alopias pelagicus* (Marshall *et al.* 2012), which are considered particularly susceptible to the stress of capture.

Post-release mortality may be high for some of these species. In Exuma Sound, The Bahamas, *Centrophorus* sp. exhibited an 83% post-release mortality rate in the first 24 h following longline capture after a mean soak time of 3.5 h (Talwar *et al.* 2017). Blood chemistry data from this study support that finding; the mean moribund blood lactate (11.16 mmol/L) and pH (7.17) values for *C. uyato* in this study were similar to the at-vessel blood lactate (9.29 mmol/L) and pH values (7.14) of those animals. Satellite telemetry data also suggest high post-release mortality for longline-caught *C. uyato* in the northern GoM (~29°N latitude; Grubbs, unpublished data) and for *Centrophorus* spp. in The Bahamas (Brooks *et al.* 2015). Using a predictive equation for post-release mortality based on lactate, glucose, and total length of longline-caught *S. cubensis* (Talwar *et al.* 2017), we suggest a higher post-release mortality rate for individuals caught in this study (73.4%) than in The Bahamas (49.7%). This difference is probably because of our catch being composed of smaller animals (mean TL: 47 cm here, 58 cm there) caught on longlines with slightly longer mean soak times (3.93 h here, 3.5 h there), which could influence higher blood lactate values (10.35 mmol/l here, 9.8 mmol/l there).

### Inter-specific differences

Blood glucose, lactate and pH values were similar in the two *Mustelus* species and *S. cubensis* and indicated a more pronounced secondary physiological stress response in those species than in *S. clarkae*, the two *Centrophorus* species and *H. griseus*. When investigating the three pairs of congeners independently, there were no significant differences in the majority of the physiological parameters assessed between *Mustelus* congeners and *Centrophorus* congeners; however, there was always a significant difference between *Squalus* congeners. The similarities between the *Centrophorus* congeners, *S. clarkae* and *H. griseus* could be attributed to their taxonomic relatedness as the *Centrophorus* congeners and*S. clarkae* are members of the same order, Squaliformes, and they are all members of the same superorder as *H. griseus*, Squalomorphii (Naylor *et al.* 2012)*.* The two *Mustelus* congeners are members of the order Carcharhiniformes, which are more distantly related to the *Centrophorus* and *Squalus* congeners and *H. griseus*, and are members of a different superorder, Galeomorphii (Naylor *et al.* 2012)*.* Interestingly, the blood chemistry profiles of the two *Squalus* congeners were different, with greater relative physiological disturbance in *S. cubensis* than *S. clarkae*, and similar blood chemistry values were observed between *S. cubensis* and *M. canis* despite being distantly related evolutionarily. While all congener pairs inhabit statistically different depths, the difference in depth between the two *Squalus* species is the greatest and may demarcate a difference between the shallower- and deeper-dwelling sharks in this study—those shallower than 300 m that inhabit the continental shelf edge and upper slope and those deeper than ~ 500 m that inhabit the mid-continental slope. With this difference in depth comes a difference in average bottom temperature, with significantly warmer and fluctuating temperatures in the shallower habitats that receive mixed epipelagic water and experience seasonality, and the relatively aseasonal, permanently cold temperatures in the deeper habitats. Furthermore, Larsen *et al.* (2020) found deeper-dwelling sharks had smaller relative heart size resulting in lower metabolic capacity than shallower-dwelling species. These results suggest that differences observed in at-vessel blood chemistry parameters between the *Mustelus* congeners and *S. cubensis* versus the remaining Squalomorphii may be driven by habitat use, rather than evolutionary history.

While individual capture duration was not measured in this study, we do know that the deeper-dwelling species experienced longer total time on hook than shallow-dwelling species after consistent soak times on bottom as it took longer to retrieve gear from deeper depths. While we do not suggest the time between hooking and gear retrieval is non-stressful, the process of retrieval from deep depths, sometimes greater than 1000 m, likely induces significant stress relative to the hooking event itself. Furthermore, only one species investigated herein, *H. griseus*, is an obligate ram ventilator, meaning that the other species are capable of lying motionless and breathing normally once hooked. Benthic-associated buccal/spiracular pumping species like those herein exhibit more subdued responses to longline capture than more active ram ventilators (Talwar *et al.* 2020), and resting on the bottom while hooked can mitigate the effects of capture on physiological disturbance (Guida *et al.* 2016; Bouyoucos *et al.* 2018).

However, even when considering the stress of retrieval, we see a greater magnitude of acidosis in the shallower-dwelling *S. cubensis* than the deeper-dwelling *S. clarkae*, for instance. One possible explanation for this could be the difference in capture temperature because of depth, with warmer capture temperatures at shallower depths driving a more pronounced response to capture. Previous studies examining capture stress in elasmobranchs have found that capture temperature may have an effect on blood chemistry. For example, blood glucose and plasma lactate were positively related to sea surface temperature at the time of hook-and-line capture in Atlantic sharpnose sharks *Rhizoprionodon terraenovae* (Hoffmayer*et al.* 2012). Similarly, in longline-captured nurse sharks, both blood glucose and plasma potassium concentrations were positively related to sea surface temperature (Bouyoucos*et al.* 2018). In trawl-captured little skates, elevations in plasma lactate and ions (Na^+^, Cl^−^, Ca^2+^, Mg^2+^, K^+^) were more extreme after aerial exposure during the summer than during the winter (Cicia *et al.* 2012). While it appears that at-vessel blood chemistry in the seven species of sharks examined herein is species specific, there are similarities between closely related species and, simultaneously, the depth and temperatures at which they inhabit may be strong drivers of their physiological response.

### Change in core temperature

When an ectothermic fish is hauled to the surface after capture, their body temperature begins to adjust to the ambient temperature, with temporary insulation from this change corresponding to the amount of muscle tissue on their body (Stevens and Sutterlin 1976). Rapid alterations in body temperature can trigger a stress response (Crawshaw 1979). In this study, the difference between the bottom temperature and sea surface temperature in the GoM was as much as 15°C at depths of 200–300 m and 25°C at depths greater than 1000 m. The subsequent change in core temperature appeared to affect at-vessel blood chemistry most prominently in the two smallest species examined, *S. cubensis* and *S. clarkae*, as well as the largest species examined, *H. griseus*. The average ΔCT was greatest in the two *Squalus* species, likely because their body sizes were the smallest. Significant changes in at-vessel blood pH were observed in both *Squalus* species as a function of ΔCT, but glucose only significantly increased with increasing ΔCT in *S. clarkae*. Changes in an ectotherm’s core body temperature can affect its metabolic rate, and, in smaller bodied ectotherms that are not able to insulate as well against this change (Stevens and Sutterlin 1976), this may result in a quicker change in metabolic rate and subsequently a greater observed stress response (Hopkins and Cech 1994; Morgan and Burgess 2007; Guida *et al.* 2016). Talwar *et al.* (2017) did not find a significant difference in many of the blood chemistry parameters examined between *S. cubensis* and *Centrophorus* sp*.*; however, their sharks were hauled to the surface at a rate of 0.3 m s^−1^ from an average depth of 760 m, while our sharks were hauled at an approximate rate of 1 m s^−1^ from a mean depth of 711 m or 535 m (depending on species). Because their sharks were hauled at a slower rate and deeper depth (but similar temperature) there was likely more time for *Centrophorus* sp*.* core temperature to warm, potentially resulting in an increased metabolic rate and subsequently a greater physiological disturbance, unlike in our study where sharks were hauled much more rapidly, not allowing the larger-bodied, deeper-dwelling sharks to warm as much as the shallower species. This also is corroborated by the greater metabolic stress observed in the *Centrophorus* sp*.* examined in Talwar *et al.* (2017) versus this study.

The significant changes observed in *H. griseus* potassium and hematocrit with increasing ΔCT are interesting and contradictory to the interpretation of the results in the two *Squalus* species; however, it should be noted that the temperature probe may not have been long enough to assess a true core temperature, and the sample size for the change in core analyses in *H. griseus* was only five sharks. Future work using a longer temperature probe and larger sample size is warranted. Furthermore, when using the Bonferroni correction $\alpha$, no significant changes were detected in stress physiology parameters for *H. griseus*.

### Capture method

In *S. cubensis*, longline capture induced higher at-vessel blood lactate than trap capture; in *C. uyato*, trap capture induced higher potassium than longline capture. The remaining blood chemistry parameters were not significantly different between capture methods. The chevron traps that were used measured 60 cm×60 cm×38 cm, and the average total length of the sharks captured in the traps was 48 and 68 cm for *S. cubensis* and *C. uyato*, respectively. Given the relatively small size of *S. cubensis*, this species was likely still able to move easily once in the trap. *S. cubensis* have been observed swimming in similar deep enclosures while ventilating normally and resting on the bottom (Talwar *et al.* 2017). As *C. uyato* is larger in size, this species may have bumped the edges of the trap more frequently than *S. cubensis*, potentially eliciting the more elevated levels of blood potassium. In past research, *Centrophorus sp.* has exhibited abnormal swimming behavior inside deep enclosures and may thus respond negatively to trap capture in a similar manner (Talwar *et al.* 2017). Regardless, no significant differences in pH, which can be a proxy for respiratory stress, were noted between the two capture methods for this species, most likely because they are able to respire without moving, and there would be no respiratory impediment in either scenario. *S. cubensis* exhibited higher at-vessel blood lactate after longline capture, probably because hooking induced a greater, and earlier, fight-or-flight response than trap capture because of the physical injury from the hook going through the fish’s jaw. Trap capture, in comparison, may not elicit stress until gear retrieval. Furthermore, the traps were typically 10% full or less by volume at retrieval, which may have reduced the incidence of physical trauma, which has been attributed to stress in trap-captured fish (Davis 2002). While we could not identify any studies that compared the stress response in fish between longline and trap capture, Hopkins and Cech (1992) did find a more pronounced stress response in gillnet-captured striped bass, *Morone saxatilis*, than those captured in traps. In elasmobranchs, gillnet capture is more stressful than longline capture (Manire *et al.* 2001; Hyatt *et al.* 2012; Guida *et al.* 2016; Prohaska *et al.* 2018). Here, it appears that in the smaller-bodied *S. cubensis*, longline capture was more stressful than trap capture, probably because of the hooking injury and the resulting behavioral and physiological response, whereas trap capture was more stressful in the larger-bodied *C. uyato*, potentially because of more limited mobility inside the trap. The interpretation of these results should be considered with caution because of the low number of *S. cubensis* and *C. uyato* that were captured in traps for comparison with longline-captured individuals.

### Distance from DWH oil spill

Given that all sharks in this study were captured in the same manner, from the same fixed stations (Fig. 1), we investigated at-vessel blood chemistry as a function of distance from the DWH oil spill site. We know that oil exposure likely occurred at this study site between 2011 and 2014 given the analysis of PAH metabolites for sharks collected in this same survey by Leary (2015). Furthermore, while catch rates at individual sampling stations varied predictably among stations and regions, they did not vary seasonally, suggesting these taxa are non-migratory. To our knowledge, no previous studies have investigated the effects of oil exposure on the physiological metrics examined herein over this length of time as they are not well suited for this examination; however, given this unique data set and the wider context of the expeditions, we were interested in this question. Of all 61 analyses for seven blood chemistry parameters, only four relationships were found to be significant, three of which had coefficients of determination less than 0.08, indicating minor relationships, and only one of which was significant at the Bonferroni-corrected $\alpha$. That significant relationship was between glucose in *C. uyato* and distance from the DWH oil spill site; results indicated that at-vessel blood glucose was higher further away. However, this range of values was similar to that measured in *Centrophorus* sp. after longline capture in areas unaffected by oil exposure (Talwar *et al.* 2017). Overall, our results indicate that there are no biologically significant relationships between these blood chemistry metrics and distance from the DWH oil spill and the relationships that we did observe are likely because of factors other than the oil spill. These results are not surprising as sharks are highly mobile organisms that, in the absence of significant barriers, can move away from locally unsuitable habitats or environmental conditions (Schlaff *et al.* 2014). Sessile organisms, on the other hand, are unable to move long distances once settled on the substrate. In addition, the oil spill occurred in 2010, and our sampling began in 2014, so we assume a low likelihood of seeing an effect of the oil spill so long after the event occurred in the physiological markers that we investigated, which are best suited to characterize acute physiological stress. Thus, the long-term effects of the DWH oil spill are less likely to be observed in sharks’ secondary stress responses, but rather in toxicological examinations which may indicate contaminants from the oil spill entering the food web through sessile organisms, then bioaccumulating and biomagnifying in these larger mobile vertebrates (Gelsleichter *et al.* 2005).

## Conclusions

Deep-sea sharks inhabiting depths shallower than 500 m generally exhibited larger relative secondary physiological stress to capture compared to those inhabiting depths greater than 500 m. We hypothesize that this pattern could be species specific but also related to their ecology. Further, small sharks like *S. cubensis* have lower thermal inertia than large sharks, and thus temperature-related stress can be greatest for small demersal sharks captured and hauled to the surface.

## Funding

This work was supported by the Gulf of Mexico Research Initiative as part of the Deep Sea to Coast Connectivity in the Eastern Gulf of Mexico (Deep-C) Consortium and through the Florida Resources and Ecosystem Sustainability, Tourist Opportunities and Revived Economies of the Gulf Coast Act of 2012 (RESTORE Act) Center of Excellence Program (4710-1126-00-F). The corresponding author, Bianca K. Prohaska, was supported in part by the Philanthropic Educational Organization’s Scholar Award, Florida State University Biology Department’s Jack Winn Gramling Endowed Scholarship, the Florida State University’s Coastal and Marine Lab Graduate Student Scholarship and the Florida Sea Grant Guy Harvey Scholarship.

## Appendix 1

Stress plot of the NMDS, which included all of the dependent variables regardless of the species. The dependent variables were at-vessel blood glucose, lactate, pH, potassium and hematocrit.

## Appendix 2

NMDS plot color coded by species; however, not analyzed by species. This included all of the dependent variables regardless of the species, which were glucose, lactate, pH, potassium and hematocrit.
